# Supplementary material for: Enhanced role of the entorhinal cortex in adapting to increased working memory load
Source: Nat Commun. 2025 Jul 1;16:5798. doi: 10.1038/s41467-025-60681-w (PMC12217308; doi:10.1038/s41467-025-60681-w)
Supplement: Supplementary file 1 — Supplementary Information [file 41467_2025_60681_MOESM1_ESM.pdf]

Supplementary information

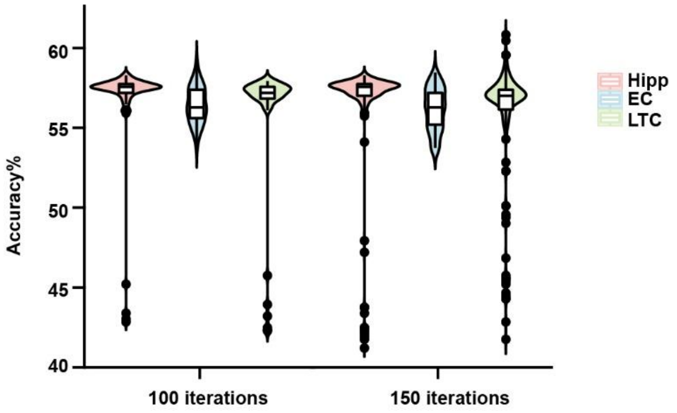

Supplementary Fig. S1 Data distribution of single-regional decoding accuracy. Left: Decoding accuracy when using power features of the hippocampus (Hipp), entorhinal cortex (EC), and lateral temporal cortex (LTC) to decode load 4 vs load 6 with 100 iterations (n = 100). Right: Decoding accuracy when using power features of the hippocampus, EC, and LTC to decode load 4 vs load 6 with 150 iterations (n = 150). Center line represents the median, and edges of the box correspond to the lower and upper quartiles, respectively. The whiskers extend to the minimum and maximum data points at most 1.5 times the interquartile range. Source data are provided as a Source Data file.

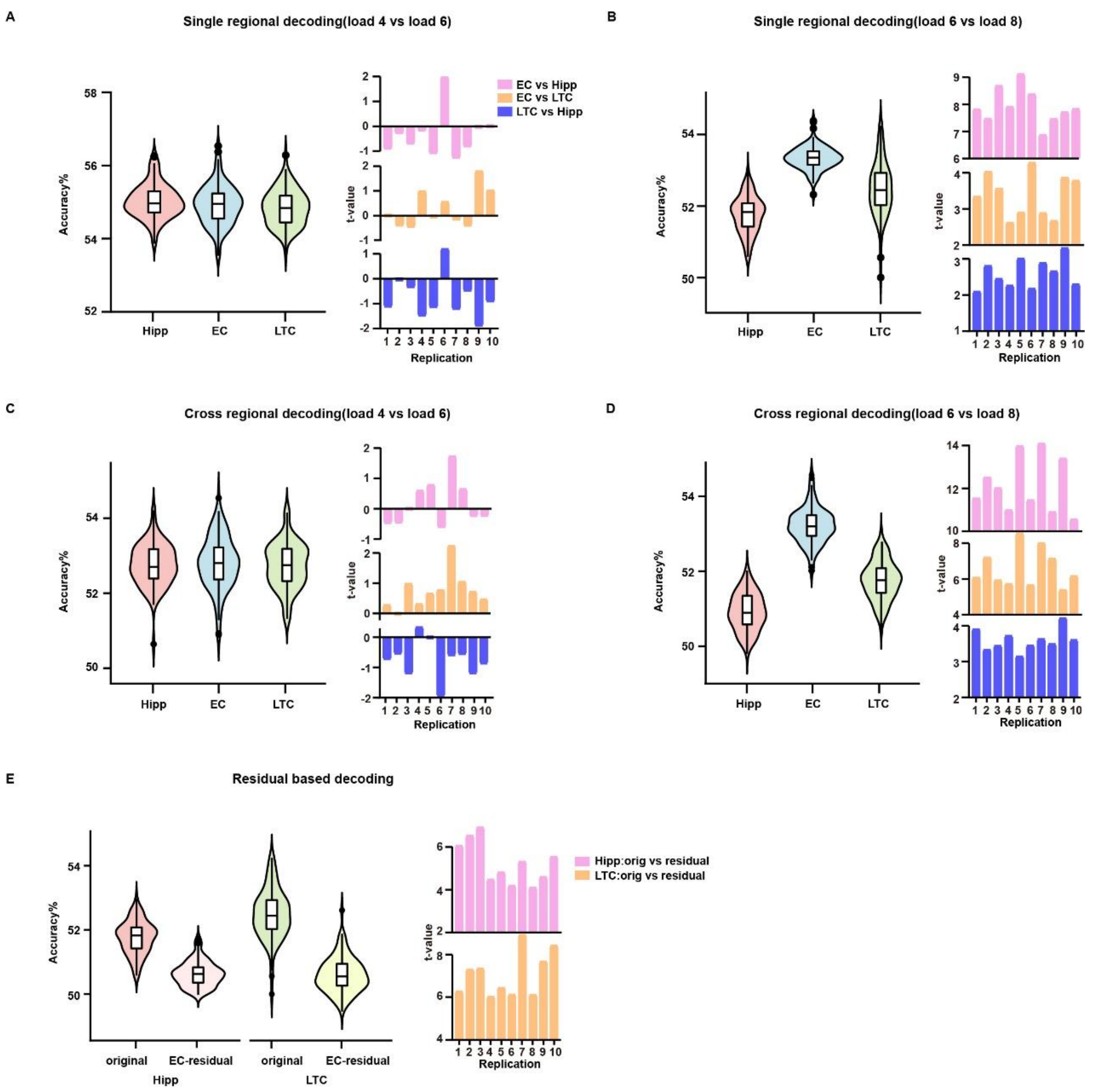

Supplementary Fig. S2 Decoding results with balanced trial counts. (A) Left: Mean single-regional decoding accuracy on low-to-medium load from the hippocampus (Hipp, light red), entorhinal cortex (EC, light blue), and lateral temporal cortex (LTC, light green) across 10 resampling iterations (n = 100 cross-validations). Right: Distribution of t-values for pairwise comparisons across brain regions (n = 10 resampling iterations; two-sided permutation t-test: EC vs hippocampus: p = 0.975; EC vs LTC: p = 0.987; LTC vs hippocampus: p = 0.988). (B) Left: Mean single-regional decoding accuracy on medium-to-high load across 10 resampling iterations (n = 100 cross-validations). Right: Distribution of t-values for pairwise comparisons across brain regions (n = 10 resampling iterations; two-sided permutation t-test: EC vs hippocampus: p < 0.001; EC vs LTC: p = 0.011; LTC vs hippocampus: p = 0.039). (C) Left: Mean cross-regional decoding accuracy on low-to-medium load across 10 resampling iterations (n = 100 cross-validations). Right: Distribution of t-values for pairwise comparisons across brain regions (n = 10 resampling iterations; two-sided permutation t-test: EC vs hippocampus: p = 0.984; EC vs LTC: p = 0.976; LTC vs hippocampus: p = 0.993). (D) Left: Mean cross-regional decoding accuracy on medium-to-high load across 10 resampling

iterations (n = 100 cross-validations). Right: Distribution of t-values for pairwise comparisons across brain regions (n = 10 resampling iterations; two-sided permutation t-test: EC vs hippocampus: $p < 0.001$; EC vs LTC: $p < 0.001$; LTC vs hippocampus: $p = 0.002$). (E) Left: Decoding accuracy using original features (dark color) and EC-residual features (light color) across 10 resampling iterations (n = 100 cross-validations). Right: Distribution of t-values comparing original (orig) and EC-residual (residual) decoding accuracy (n = 10 resampling iterations; two-sided permutation t-test: all *ps* < 0.001). The p-values reported above correspond to the maximum values obtained across 10 resampling iterations. Center line represents the median, and edges of the box correspond to the lower and upper quartiles, respectively. Whiskers extend to the minimum and maximum data points at most 1.5 times the interquartile range. Source data are provided as a Source Data file.

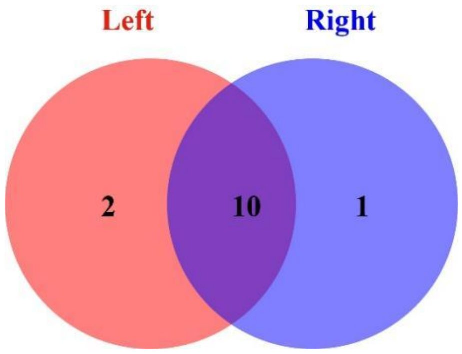

Supplementary Fig. S3 EC Electrode Placement across Hemispheres. The Venn diagram shows the distribution of participants with EC electrodes: 2 had electrodes only in the left EC, 10 had electrodes in both hemispheres' EC, and 1 had an electrode only in the right EC. Source data are provided as a Source Data file.

Supplementary Table S1. Subject characteristics.

Patient	Sex	Pathology	Seizure onset zone	
1	f	Xanthoastrozytoma WHO II	Hippocampus	right
2	m	Hippocampal gliosis	Hippocampus	right
3	f	Hippocampal sclerosis	Hippocampus	left
4	m	Posttraumatical lesion	Hippocampus	bilateral
5	m	Hippocampal sclerosis	Amygdala	right
6	m	Hippocampal sclerosis	Hippocampus	right
7	f	mesial temporal sclerosis	Entorhinal cortex	right
8	f	mesial temporal sclerosis	Amygdala	right
9	m	non-lesional	Hippocampus	bilateral
10	f	Hippocampal sclerosis	Hippocampus	left
11	m	Dysembryoplastic neuroepithelial tumour	Hippocampus	left
12	f	non-lesional	Hippocampus	bilateral
13	m	Hippocampal sclerosis	Hippocampus	bilateral

Supplementary Table S2. Chance level for all decoding analyses.

		EC	Hippocampus	LTC
**Single-regional (%)**	**load 4 vs load 6**	53.16	52.37	50.37
	**load 6 vs load 8**	51.05	50.73	51.02
**Cross-regional (%)**	**load 4 vs load 6**	53.27	50.42	57.25
	**load 6 vs load 8**	50.07	50.21	50.63
**Residuals-based (%)**	**EC-resudual**	/	50.02	50.11
	**Control analysis**	/	50.31	50.05
**Single-regional groups (%)**	**low group**	50.24	50.98	50.57
	**high group**	50.13	50.20	50.18
**Cross-regional groups (%)**	**low group**	50.29	50.16	50.48
	**high group**	50.12	50.12	50.14

Supplementary Table S3. Decoding accuracy with 50, 100, and 150 iterations and cross-validations. Each decoding result presented the mean accuracy with the corresponding chance level (mean accuracy ± SD [chance level]).

			50 times	100 times	150 times
Single-regional (%)	load 4 vs load 6	EC	56.45 ± 1.14 [52.88]	56.44 ± 1.10 [53.16]	56.35 ± 1.29 [52.87]
		Hippocampus	56.34 ± 3.84 [52.26]	56.33 ± 3.94 [52.37]	56.25 ± 4.42 [50.32]
		LTC	56.17 ± 3.69 [50.45]	56.08 ± 3.84 [50.37]	56.16 ± 3.92 [52.34]
	load 6 vs load 8	EC	55.43 ± 1.83 [51.05]	55.49 ± 1.72 [51.05]	55.62 ± 1.75 [51.04]
		Hippocampus	52.85 ± 1.70 [50.95]	52.84 ± 1.67 [50.73]	52.89 ± 2.28 [50.70]
		LTC	54.00 ± 2.29 [51.02]	53.82 ± 2.10 [51.02]	53.81 ± 1.91 [51.01]
Cross-regional (%)	load 4 vs load 6	EC	57.14 ± 1.24 [52.96]	57.25 ± 1.14 [53.27]	57.21 ± 1.26 [52.63]
		Hippocampus	57.06 ± 1.63 [50.68]	57.14 ± 1.83 [50.42]	57.19 ± 0.41 [50.37]
		LTC	57.65 ± 1.26 [57.30]	57.51 ± 1.47 [57.25]	57.50 ± 1.02 [57.21]
	load 6 vs load 8	EC	53.07 ± 2.63 [50.15]	53.13 ± 2.61 [50.07]	53.07 ± 1.25 [49.99]
		Hippocampus	50.47 ± 2.12 [50.29]	50.45 ± 2.25 [50.21]	50.61 ± 1.57 [50.16]
		LTC	51.89 ± 1.75 [50.71]	51.97 ± 1.97 [50.63]	51.96 ± 1.37 [50.67]
Residuals-based (%)	EC-resudual	Hippocampus	50.38 ± 1.40 [50.11]	50.34 ± 1.33 [50.02]	50.34 ± 1.14 [49.99]
		LTC	50.40 ± 1.1 [50.16]	50.31 ± 1.41 [50.11]	50.42 ± 1.56 [50.37]
	Control analysis	Hippocampus	51.30 ± 0.94 [50.38]	51.36 ± 1.22 [50.31]	51.29 ± 1.08 [50.26]
		LTC	50.96 ± 1.52 [50.05]	50.78 ± 1.48 [50.05]	51.70 ± 1.47 [50.76]
Single-regional groups (%)	low group	EC	53.19 ± 3.00 [50.26]	53.21 ± 3.24 [50.24]	53.37 ± 3.19 [50.26]
		Hippocampus	53.91 ± 3.20 [51.18]	53.89 ± 3.16 [50.98]	54.08 ± 3.08 [50.16]

		LTC	52.20 ± 3.37 [50.67]	52.18 ± 3.47 [50.57]	52.05 ± 3.18 [50.58]
	**high group**	EC	56.24 ± 2.67 [50.25]	56.22 ± 2.87 [50.13]	56.31 ± 2.68 [50.11]
		Hippocampus	54.04 ± 3.75 [50.30]	54.12 ± 3.32 [50.20]	54.16 ± 3.43 [50.97]
		LTC	51.74 ± 4.66 [50.32]	51.76 ± 4.60 [50.18]	51.64 ± 4.39 [50.15]
**Cross-regional groups (%)**	**low group**	EC	52.64 ± 2.24 [50.42]	52.57 ± 2.17 [50.29]	52.62 ± 2.09 [50.22]
		Hippocampus	51.43 ± 2.96 [50.24]	51.53 ± 2.81 [50.16]	51.49 ± 2.18 [50.25]
		LTC	51.58 ± 2.58 [50.62]	51.64 ± 2.72 [50.48]	51.57 ± 2.15 [50.42]
	**high group**	EC	54.40 ± 2.14 [50.19]	54.39 ± 2.33 [50.12]	54.42 ± 1.99 [50.10]
		Hippocampus	51.89 ± 2.27 [50.14]	51.95 ± 2.46 [50.12]	51.85 ± 2.02 [50.12]
		LTC	51.39 ± 2.24 [50.24]	51.36 ± 2.13 [50.14]	51.32 ± 2.09 [50.16]
