# Supplementary material for: Enhanced role of the entorhinal cortex in adapting to increased working memory load
Source: Nat Commun. 2025 Jul 1;16:5798. doi: 10.1038/s41467-025-60681-w (PMC12217308; doi:10.1038/s41467-025-60681-w)
Supplement: Supplementary file 2 — Reporting Summary [file 41467_2025_60681_MOESM2_ESM.pdf]

nature portfolio

Corresponding author(s): Jin Li, Dan Cao

Last updated by author(s): May 7, 2025

# Reporting Summary

Nature Portfolio wishes to improve the reproducibility of the work that we publish. This form provides structure for consistency and transparency in reporting. For further information on Nature Portfolio policies, see our Editorial Policies and the Editorial Policy Checklist.

## Statistics

For all statistical analyses, confirm that the following items are present in the figure legend, table legend, main text, or Methods section.

| n/a | Confirmed | |
|---|---|---|
| ☐ | ☒ | The exact sample size (*n*) for each experimental group/condition, given as a discrete number and unit of measurement |
| ☐ | ☒ | A statement on whether measurements were taken from distinct samples or whether the same sample was measured repeatedly |
| ☐ | ☒ | The statistical test(s) used AND whether they are one- or two-sided<br>*Only common tests should be described solely by name; describe more complex techniques in the Methods section.* |
| ☒ | ☐ | A description of all covariates tested |
| ☐ | ☒ | A description of any assumptions or corrections, such as tests of normality and adjustment for multiple comparisons |
| ☐ | ☒ | A full description of the statistical parameters including central tendency (e.g. means) or other basic estimates (e.g. regression coefficient) AND variation (e.g. standard deviation) or associated estimates of uncertainty (e.g. confidence intervals) |
| ☐ | ☒ | For null hypothesis testing, the test statistic (e.g. $F$, $t$, $r$) with confidence intervals, effect sizes, degrees of freedom and $P$ value noted<br>*Give P values as exact values whenever suitable.* |
| ☒ | ☐ | For Bayesian analysis, information on the choice of priors and Markov chain Monte Carlo settings |
| ☒ | ☐ | For hierarchical and complex designs, identification of the appropriate level for tests and full reporting of outcomes |
| ☒ | ☐ | Estimates of effect sizes (e.g. Cohen's *d*, Pearson's *r*), indicating how they were calculated |

*Our web collection on statistics for biologists contains articles on many of the points above.*

## Software and code

Policy information about availability of computer code

| | |
|---|---|
| Data collection | Neurophysiological data were collected using the ATLAS system (Neuralynx Inc., Bozeman, Montana, USA). See detailed description in the Methods section. |
| Data analysis | Data analyses were performed using Matlab(version R2019b),FieldTrip Toolbox(20190527),LIBSVM package(libsvm-3.32)and EEGLAB toolbox(eeglab 14_1_0b).Anatomicaol data analysis was performed using Fieldtrip Toolbox 20190527.The brain figures were visualized by BrainNet Viewer toolbox.The custom code supporting this study is available at https://doi.org/10.5281/zenodo.15355761. |

For manuscripts utilizing custom algorithms or software that are central to the research but not yet described in published literature, software must be made available to editors and reviewers. We strongly encourage code deposition in a community repository (e.g. GitHub). See the Nature Portfolio guidelines for submitting code & software for further information.

## Data

Policy information about availability of data

All manuscripts must include a data availability statement. This statement should provide the following information, where applicable:

- Accession codes, unique identifiers, or web links for publicly available datasets
- A description of any restrictions on data availability
- For clinical datasets or third party data, please ensure that the statement adheres to our policy

The raw data generated in this study have been deposited in the public database under accession link https://doi.gin.g-node.org/10.12751/g-node.d76994/.

## Research involving human participants, their data, or biological material

Policy information about studies with human participants or human data. See also policy information about sex, gender (identity/presentation), and sexual orientation and race, ethnicity and racism.

| | |
|---|---|
| Reporting on sex and gender | Thirteen subjects participated in this study, including six females and seven males. Sex and gender were not variables in our study. Sex(as recorded by clinical centers) is reported in the demographic information provided in Supplementary Table S1. |
| Reporting on race, ethnicity, or other socially relevant groupings | Race, ethnicity, and other socially relevant groupings were not reported in this manuscript. |
| Population characteristics | Thirteen subjects (six females; mean age ± SD: 35 ± 13 years) participated in this study. |
| Recruitment | Data were collected at the Swiss Epilepsy Center,Klinik Lengg, Switzerland.Subjects undergoing invasive electrophysiological recording for clinical purposes and having channels in the hippocampus, entorhinal cortex, and lateral temporal cortex in the same hemisphere were included in the current study. All participation in the study was voluntary. |
| Ethics oversight | The study was approved by the local ethics committee (Kantonale Ethikkommission Zürich, PB 2016-02055), and all subjects gave written informed consent. |

Note that full information on the approval of the study protocol must also be provided in the manuscript.

# Field-specific reporting

Please select the one below that is the best fit for your research. If you are not sure, read the appropriate sections before making your selection.

☒ Life sciences ☐ Behavioural & social sciences ☐ Ecological, evolutionary & environmental sciences

For a reference copy of the document with all sections, see nature.com/documents/nr-reporting-summary-flat.pdf

# Life sciences study design

All studies must disclose on these points even when the disclosure is negative.

| | |
|---|---|
| Sample size | We determined the sample size based on previous iEEG studies.Our study aimed to obtain at least 10 subjects with electrodes in the hippocampus,entorhinal cortex,and lateral temporal cortex. Our sample sizes(N=13)are similar to iEEG studies in the field. |
| Data exclusions | We excluded 140/3057 trials due to artifacts, typically caused by large singular artifacts from cable movement or signal drift. |
| Replication | The experimental procedure was repeatd 2 to 8 times (on average 5 times) with 13 different participants. The analyses were performed at local field potential level. The effect reported in the study were consistent and replicated. |
| Randomization | In this study, we employed a within-subjects analysis, whereby each participant was tested in the same analysis set and had all types of trials. We performed permutation testing where appropriate to ensure statistical validity of our results. |
| Blinding | Subjects were not aware of the specific goals of the study. There was no subjective measurement or decision that the investigator needed to make during the experiment. All the data are collected and analyzed off-line. Data collection and analysis were not performed blind to the conditions of the experiments as conditional information is required for further analyses. |

# Reporting for specific materials, systems and methods

We require information from authors about some types of materials, experimental systems and methods used in many studies. Here, indicate whether each material, system or method listed is relevant to your study. If you are not sure if a list item applies to your research, read the appropriate section before selecting a response.

## Materials & experimental systems

| n/a | Involved in the study | |
|---|---|---|
| ☒ | ☐ | Antibodies |
| ☒ | ☐ | Eukaryotic cell lines |
| ☒ | ☐ | Palaeontology and archaeology |
| ☒ | ☐ | Animals and other organisms |
| ☒ | ☐ | Clinical data |
| ☒ | ☐ | Dual use research of concern |
| ☒ | ☐ | Plants |

## Methods

| n/a | Involved in the study | |
|---|---|---|
| ☒ | ☐ | ChIP-seq |
| ☒ | ☐ | Flow cytometry |
| ☒ | ☐ | MRI-based neuroimaging |

## Plants

| | |
|---|---|
| Seed stocks | N/A |
| Novel plant genotypes | N/A |
| Authentication | N/A |
